# Does a New Antibiotic Scheme Improve the Outcome of *Staphylococcus aureus*-Caused Acute Prosthetic Joint Infections (PJI) Treated with Debridement, Antibiotics and Implant Retention (DAIR)?

**DOI:** 10.3390/antibiotics11070922

**Published:** 2022-07-08

**Authors:** Álvaro Auñón, Miguel Tovar-Bazaga, Antonio Blanco-García, Joaquín García-Cañete, Raúl Parrón, Jaime Esteban

**Affiliations:** 1 Department of Orthopedic Surgery, IIS-Fundación Jiménez Díaz, CIBERINFEC, CIBER de Enfermedades Infecciosas, 28040 Madrid, Spain; raul.parron@quironsalud.es; 2 Department of Orthopedic Surgery, Hospital Universitario Fundación Jiménez Díaz, 28040 Madrid, Spain; miguel.tovar@quironsalud.es; 3 Department of Internal Medicine, IIS-Fundación Jiménez Díaz, CIBERINFEC, CIBER de Enfermedades Infecciosas,28040 Madrid, Spain; ablancog@fjd.es (A.B.-G.); jgarciac@fjd.es (J.G.-C.); 4 Department of Clinical Microbiology, IIS-Fundación Jiménez Díaz, CIBERINFEC, CIBER de Enfermedades Infecciosas, 28040 Madrid, Spain; jestebanmoreno@gmail.com

**Keywords:** *Staphylococcus aureus*, prosthetic joint infection, debridement, antibiotics and implant retention, daptomycin, cloxacillin, biofilm

## Abstract

One of the most commonly used treatments for acute prosthetic joint infection (PJI) is DAIR (debridement, antibiotics and implant retention), which comprises the debridement and the retention of the implant, followed by antibiotic treatment. The efficacy of DAIR remains unclear, as the literature has demonstrated variable success rates, ranging from 26% to 92%. The *Staphylococcus aureus* is one of the most closely related causative microorganisms, especially with acute and late-acute PJI; it has been identified as one of the most significant predictors of DAIR failure. The current guidelines consider the use of vancomycin as the therapy of choice, but it requires the close control of possible side effects. The aim of this study is to determine if a new combination of antibiotics (a highly bactericidal initial combination followed by an antibiofilm scheme) decreases the failure of DAIR-treated acute prosthetic joint infection (PJI) caused by *Staphylococcus aureus.* A retrospective analysis of cases of orthopedic infections during a nine-year period (2011–2019) was performed. A total of 45 acute PJI cases caused by *S. aureus* were diagnosed. The results of two antibiotic schemes were compared: a novel scheme comprising 5 days of daptomycin (10 mg/kg/24 h) + cloxacillin (2 g/6 h) followed by levofloxacin (500 mg/24 h) + rifampicin (600 mg/24 h), versus a traditional, less bactericidal scheme of vancomycin (1000 mg/12 h) plus rifampicin (600 mg/24 h) or levofloxacin (500 mg/24 h) plus rifampicin (600 mg/24 h). Twenty-two out of the twenty-four patients treated with the new scheme (91.6%) were free of infection after 24.8 months of mean follow-up, whereas fourteen out of twenty-one patients (66.6%) were free of infection after 46.6 months of follow-up. This difference was statistically significant (*p* = 0.036). Demographic comparisons demonstrated homogeneous features, except the Charlson score, which was higher in the novel scheme group (*p* = 0.047). The combination of high-dose daptomycin and cloxacillin, followed by levofloxacin plus rifampicin, together with surgical treatment, shows better results when compared with other antibiotic schemes for treating acute PJI caused by *S. aureus* in which DAIR was performed.

## 1. Introduction

The use of prosthetic joint implants is a common procedure in orthopedic surgery. According to some reports, there is a projected increase in the number of joint replacements, so it is expected that prosthetic joint infections (PJI) will grow as well, since current statistics show infection rates of 1.0% for hip and 0.5% to 2% for knee [1].

One of the most used treatments for acute PJI is DAIR (debridement, antibiotics, and implant retention), which comprises the debridement and retention of the implant, followed by antibiotic treatment. The efficacy of DAIR remains unclear, as the literature has demonstrated variable success rates, ranging from 26% to 92% [2].

Regarding PJI, *Staphylococcus aureus* is one of the most closely related causative microorganisms, especially with acute and late–acute PJI [3]. *S. aureus* has been identified as the one of most significant predictors of DAIR failure [4,5,6,7,8], suggesting a prominent role of extrapolymeric biofilm substance in the infectious burden. Methicillin-resistant *S. aureus* (MRSA) and methicillin-susceptible *S. aureus* (MSSA) show similar treatment failure rates, although the timing of failure varies; in MRSA, PJI frequently occurs in the first weeks after debridement, while patients are still on the therapy; in contrast, half of MSSA failures occur once the antibiotic treatment has been withdrawn [9].

The current guidelines consider the use of vancomycin as the therapy of choice, but it requires the close control of possible side effects [10]. As an alternative to vancomycin, daptomycin could be used. Daptomycin is a cyclic lipopeptide antibiotic that has been largely used in the last decade for difficult-to-treat gram-positive infections [11], mostly bacteriemia or endocarditis [12,13,14]. It shows a high bactericidal effect, as well as antibiofilm capacity and intraosteoblastic activity, when used in combination with oxacillin [15]. Furthermore, in vitro studies have shown that the development of daptomycin resistance is usually accompanied by a concomitant decrease in oxacillin resistance, enhancing its bactericidal effect in what has been termed a “seesaw” effect [16]. Considering these data, we defined the ideal antibiotic scheme as the one which provides an initial highly bactericidal combination, followed by an antibiofilm oral combination, if possible. It should provide wide-spectrum benefits, the acquisition of a synergistic effect, and a low risk of the emergence of drug-resistant strains [17].

The aim of this study was to review our experience and to compare the results of the new antibiotic scheme with an initial highly bactericidal activity, followed by a combination with antibiofilm activity, with the combinations previously used for the treatment of acute PJI caused by *S. aureus* in which DAIR was performed.

## 2. Results

### 2.1. Demographic Results

Forty-five cases of acute PJI by *S. aureus* were diagnosed between 2011 and 2019 according to positive PJI ICM-criteria. The average age of patients at diagnosis was 69.6 years (standard deviation (SD): 12.5; range: 44–94); 27 were males (60%) and 18 females (40%), with a mean Charlson score of 4.06 (SD 2.03; range: 0–9). The mean follow-up was 34.9 months (SD 16.05, range: 18–72) and the mean antibiotic treatment duration was 94.2 days (SD 36.5, range: 35–180).

The infected implants included 32 total hip arthroplasties (THA) (70%) and 13 total knee arthroplasties (TKA) (30%). The mean time between index surgery and DAIR was 23.7 days (SD 6.7, range: 11–38). Thirty-nine acute PJI were caused by MSSA (87%) and six were caused by MRSA (13%).

**Figure 1:**
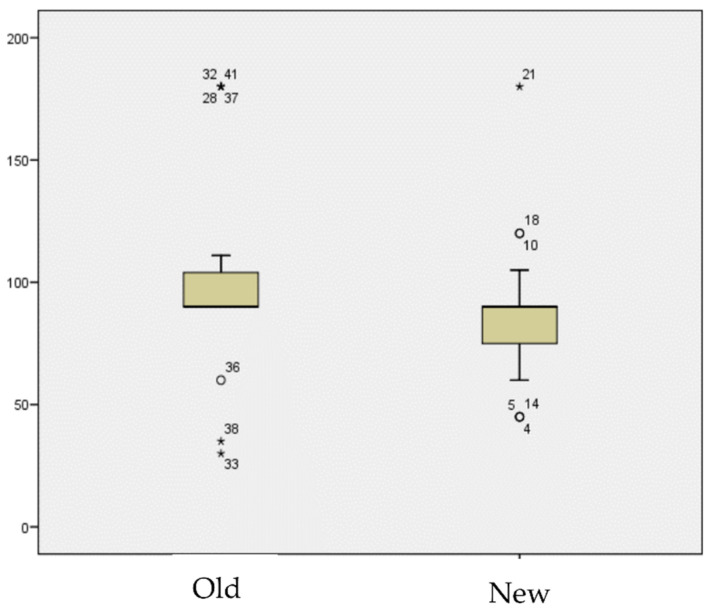
Days of treatment.

Demographic comparisons between the two groups demonstrated similar features except for the Charlson score (*p* = 0.047), which was higher in the daptomycin group (mean 4.63, SD 1.7, range: 3–9 vs. mean 3.43, SD 2.15, range: 0–8). The result is shown in Figure 1. There was no difference in terms of age (*p* = 0.645), MRSA incidence (*p* = 0.482), or time between index surgery and DAIR (*p* = 0.393). The demographic and treatment data are shown in Table 1 and Table 2.

### 2.2. Treatment Results

Twenty-four patients (53%) were treated with the new scheme (daptomycin + cloxacillin followed by levofloxacin–rifampicin) during a mean of 87.5 days (SD 28.6, range: 45–180), and twenty-one (47%) were treated with another combination (mostly comprising vancomycin or rifampicin with diverse antibiotics) during a mean of 102 days (SD 42.3, range: 35–180).

Twenty-two out of the twenty-four patients treated with the new scheme (91.6%) were free of infection after 24.8 months of mean follow-up. The two patients in whom treatment failed required further DAIR. Despite the high dose of daptomycin, no side effects were reported.

On the contrary, in the other schemes group, fourteen out of twenty-one patients (66.6%) were free of infection after 46.6 months of follow-up. Six patients underwent another DAIR, while one patient required a two-stage revision.

**Figure 2:**
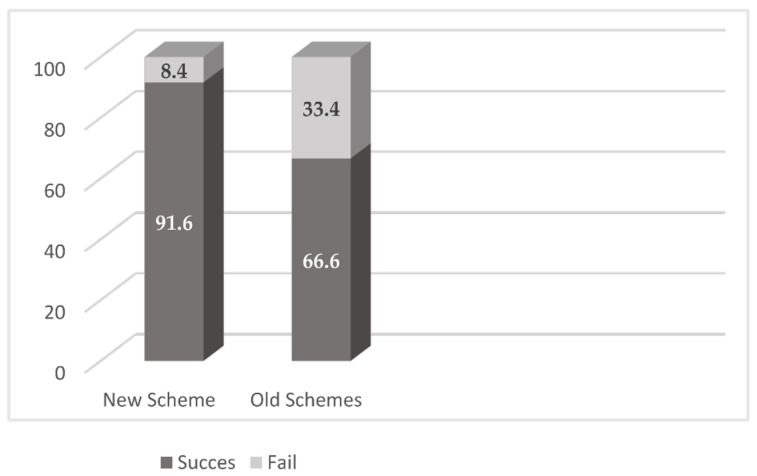
Percentage of clinical cure.

**Figure 3:**
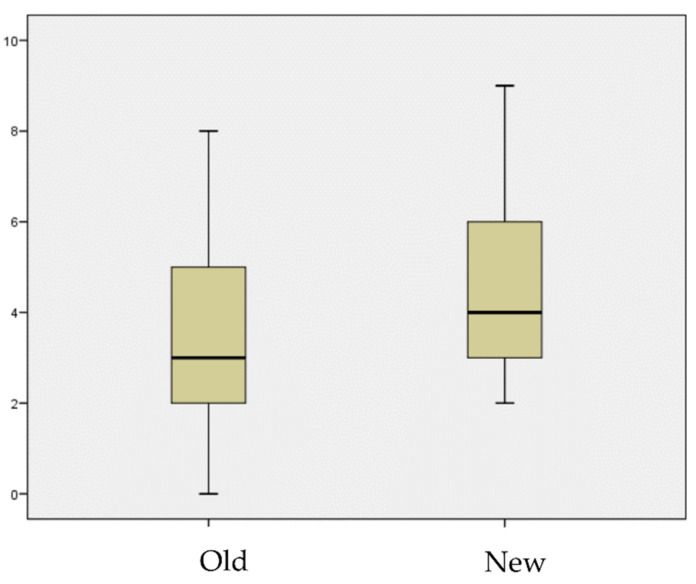
Charlson score according to each group.

This difference in the outcome was statistically significant (*p* = 0.036). There was no difference in terms of follow-up, MRSA infection rate, rifampin resistance, or duration of antibiotic treatment. These results are shown in Figure 2 and Figure 3.

## 3. Discussion

One of the most closely related microorganisms with PJI is *S. aureus*, mainly with acute and late acute PJI. The current guidelines consider vancomycin as the antibiotic therapy of choice, but it requires the close control of possible side effects [3]. The development of optimal therapeutic protocols is still a challenge. Thus, we propose to explore the effectiveness of a new antibiotic scheme, which combines an initial bactericidal regime followed by an antibiofilm one. Compared with several previous combinations, we found a clinically and statistically significant difference in favor of the novel regime even after a mean follow-up of 35 months. To date, this is the first study to analyze in vivo the synergic effect of daptomycin and oxacillin when treating PJI caused by *S. aureus* after DAIR.

There is no standardized protocol for the treatment of acute PJI caused by *S. aureus,* including the duration of treatment or antibiotic choice.

Daptomycin is an antimicrobial agent with a long half-life and the ability to treat multi-resistant gram-positive infections. Adverse effects of the prolonged use of daptomycin have been described and include the elevation of serum creatin phosphokinase (CPK), rash, eosinophilic pneumonia, or acute renal failure secondary to massive rhabdomyolysis [18], although there are some trials [19] in which doses of 6–8 mg/kg/day up to six weeks have been used for PJI in patients undergoing two-stage revisions, with great results in terms of efficacy and safety. In our study, no side effects were described. Although we used a high-dose regime, the short treatment course could explain the lack of side effects.

Some in vitro and in vivo studies [17] have shown that the combination of daptomycin and other antimicrobial agents such as fosfomycin at a dose of 10 mg/kg/day presents synergistic or additive effects against MRSA [20]. Interestingly, the combination with oxacillin showed low [17], moderate [21], or enhanced synergistic effect [22] in different studies. Moreover, the aforementioned effect has been found with other antimicrobial agents [22]. Other beneficial properties of combination regimes with daptomycin include an intraosteoblastic activity against *S. aureus* [15] and the previously mentioned “seesaw” effect [16].

The daptomycin–rifampicin combination has been used, with safe and effective results, in the treatment of bacteriemia (8 mg/kg/day) [10]. Moreover, daptomycin has also been proposed in the treatment of knee and hip periprosthetic joint infections [18,23,24,25] with a success rate between 54.5–100%, at doses between 4 mg/kg/day [25] and >6 mg/kg/day [24].

Lora-Tamayo et al. [26] described the combination of daptomycin at 10 mg/kg/day with rifampicin, for PJI treated with DAIR, with good tolerance and optimal clinical and microbiological outcomes, and a decreased failure rate; these results support our theory of improvement in the treatment of acute PJI caused by *S. aureus.* Other groups of study have also described the use of high-dose daptomycin without major side effects [27,28].

However, our study has several limitations. First, the heterogeneity of one of the groups included in the comparison, since it comprises several combinations of antibiotics. Second, we have a limited sample size. Third, this was not a clinical trial or a randomized stratified study.

In conclusion, the combination of high-dose daptomycin and cloxacillin, followed by levofloxacin plus rifampicin, together with appropriate surgical treatment, shows good results when compared with other antibiotic schemes in the treatment of acute PJI caused by *S. aureus* in which DAIR was performed.

## 4. Materials and Methods

### 4.1. Study Design, Patients and Settings

A retrospective analysis of cases of orthopedic device-related infections in our institution, a 686-bed tertiary hospital, during a nine-year period (2011–2019) was performed.

### 4.2. Data Collection

Out of 1255 bone and joint infections, 191 were diagnosed as acute PJI, and 45 of them were caused by *S. aureus*; polymicrobial infections were excluded. Clinical records were reviewed following a previously determined protocol, including time between index surgery and DAIR, antibiotic treatment scheme, and follow-up. Minimum follow-up was set at 12 months. Acute PJI cases were considered those diagnosed less than six weeks after prosthesis implantation, following a previously established protocol, which included preoperative blood tests (white blood cell counts, erythrocyte sedimentation rate, C-reactive protein), joint aspiration when possible, and microbiological cultures from 5–7 samples. The date of diagnosis was considered as the date of the first surgical intervention in which positive cultures for *S. aureus* were obtained.

### 4.3. Definition

Diagnosis of PJI was performed according to the ICM Criteria [29]. Failure was defined as lack of infection control, including persistent signs of infection (fistulae, elevated acute phase reactants), infection-attributable mortality, the need for further debridement, and the decision to opt for suppressive antibiotic therapy. Demographic data from patients and the treatment group is shown in Table 1 and Table 2.

Our institutional protocol for DAIR involves open debridement of the joint, complete synovectomy, copious pulsatile lavage, and exchange of all modular components (head/liner in total hip arthroplasty [THA]; polyethylene liner in total knee arthroplasty [TKA]).

### 4.4. Groups Division

Based on its high bactericidal effect, as well as antibiofilm capacity, we therefore chose a new scheme three years ago, comprising an initial treatment with daptomycin iv (10 mg/kg each 24 h) plus cloxacillin iv (2 g/6 h) for 5 days, followed by levofloxacin po (500 mg/24 h) plus rifampicin po (600 mg/24 h) for 90 days.

This regime was compared to the traditional approach based on the use of less-bactericidal combinations, mainly comprising vancomycin iv (1000 mg/12 h) plus rifampicin po (600 mg/24 h) or levofloxacin po (500 mg/24 h) plus rifampicin po (600 mg/24 h).

### 4.5. Statistical Analysis

Continuous variables were expressed as average, range, and median where appropriate, and categorical variables as absolute value and/or percentages of the total sample for that variable. A *p* value ≤ 0.05 was considered to indicate statistical significance, χ 2 test was used to compare percentages and Student’s *t*-test was used to compare means. Data analysis was performed using IBM^®^SPSS^®^, version 22.0. Consent to perform the study was obtained from the Research Ethics Committee of our hospital.

## 5. Conclusions

The combination of high-dose daptomycin and cloxacillin, followed by levofloxacin plus rifampicin, together with appropriate surgical treatment, improves the success rate when compared with other antibiotic schemes in the treatment of acute PJI caused by *S. aureus* in which DAIR was performed, even in more complex groups of patients.

## Figures and Tables

**Table 1:** Demographic data according to treatment group.

	Daptomycin-Cloxacillin	Other Treatments
Number of patients	24	21
Mean Age (years)	70.4	68.7
Sex (M:F)	1:1	1.6:1
Charlson	4.63	3.43
THA	6	7
TKA	18	14
Days of treatment (SD)	87.5 (28.6)	102 (42.3)
Failure	2	7
Follow-up (months)	15	31.2

Abbreviations: THA, total hip arthroplasty; TKA, total knee arthroplasty; M, male; F, female; SD, standard deviation.

**Table 2:** Treatment data from patients.

Patient	Age	Sex	Pathogen	Charlson Score	Antibiotic Scheme	Duration (Days)	Clinical Cure
1	67	F	MSSA	3	Old	90	Yes
2	65	M	MSSA	3	Old	90	No
3	55	M	MSSA	4	Old	90	Yes
4	71	M	MSSA	4	Old	180	Yes
5	75	F	MSSA	2	Old	111	Yes
6	76	M	MSSA	6	Old	90	No
7	51	M	MSSA	4	Old	90	No
8	78	F	MSSA	4	Old	180	No
9	88	M	MSSA	3	Old	30	Yes
10	49	M	MSSA	4	Old	90	Yes
11	51	M	MSSA	6	Old	90	Yes
12	66	F	MSSA	8	Old	60	Yes
13	76	M	MSSA	5	Old	180	No
14	85	F	MSSA	6	Old	35	No
15	81	F	MRSA	6	Old	90	Yes
16	67	F	MRSA	3	Old	104	Yes
17	77	F	MSSA	7	Old	180	Yes
18	44	M	MSSA	4	Old	90	Yes
19	58	M	MSSA	3	Old	90	No
20	84	M	MSSA	4	Old	90	Yes
21	78	M	MSSA	4	Old	90	Yes
22	79	M	MSSA	3	New	90	Yes
23	71	M	MSSA	9	New	90	Yes
24	67	F	MSSA	6	New	60	Yes
25	59	M	MSSA	4	New	45	Yes
26	53	M	MSSA	3	New	45	Yes
27	94	M	MSSA	1	New	90	Yes
28	73	F	MRSA	4	New	90	Yes
29	71	F	MSSA	5	New	90	Yes
30	65	F	MRSA	3	New	90	Yes
31	77	F	MSSA	2	New	120	Yes
32	83	F	MSSA	3	New	90	Yes
33	83	F	MRSA	5	New	60	Yes
34	47	M	MSSA	0	New	60	Yes
35	48	M	MSSA	8	New	45	Yes
36	78	F	MSSA	2	New	90	Yes
37	61	M	MSSA	6	New	90	No
38	79	F	MSSA	5	New	90	Yes
39	81	M	MSSA	5	New	120	No
40	62	M	MSSA	2	New	90	Yes
41	74	M	MSSA	3	New	105	Yes
42	61	M	MSSA	0	New	180	Yes
43	59	F	MSSA	1	New	90	Yes
44	80	M	MSSA	7	New	90	Yes
45	85	M	MRSA	3	New	90	Yes

Abbreviations: MSSA, methicillin-susceptible *Staphylococcus aureus; MRSA* methicillin-resistant *Staphylococcus aureus;* M, male; F, female.

## Data Availability

The data presented in this study are available on request from the corresponding author.
